# A Parallel Implementation of the Wuchty Algorithm with Additional Experimental Filters to More Thoroughly Explore RNA Conformational Space

**DOI:** 10.1371/journal.pone.0117217

**Published:** 2015-02-19

**Authors:** Jonathan W. Stone, Samuel Bleckley, Sean Lavelle, Susan J. Schroeder

**Affiliations:** 1 Department of Chemistry and Biochemistry, University of Oklahoma, Norman, Oklahoma, United States of America; 2 Department of Microbiology and Plant Biology, University of Oklahoma, Norman, Oklahoma, United States of America; Ben-Gurion University, ISRAEL

## Abstract

We present new modifications to the Wuchty algorithm in order to better define and explore possible conformations for an RNA sequence. The new features, including parallelization, energy-independent lonely pair constraints, context-dependent chemical probing constraints, helix filters, and optional multibranch loops, provide useful tools for exploring the landscape of RNA folding. Chemical probing alone may not necessarily define a single unique structure. The helix filters and optional multibranch loops are global constraints on RNA structure that are an especially useful tool for generating models of encapsidated viral RNA for which cryoelectron microscopy or crystallography data may be available. The computations generate a combinatorially complete set of structures near a free energy minimum and thus provide data on the density and diversity of structures near the bottom of a folding funnel for an RNA sequence. The conformational landscapes for some RNA sequences may resemble a low, wide basin rather than a steep funnel that converges to a single structure.

## Background

The flood of RNA sequence information from new high-throughput technologies creates a need for efficient computational tools for predicting structure and function. Predicting an RNA secondary structure is often the first step towards discovering the structure and function of a new noncoding RNA sequence. Free energy minimization is the most common approach to generate an RNA structure prediction [1,2]. The thermodynamic parameters that form the basis of most free energy minimization algorithms are constantly being updated and improved [3,4]. However, free energy minimization does not consider RNA tertiary interactions, RNA-protein interactions, kinetic traps, or cotranscriptional folding [5]. For long RNA sequences, very different secondary structures may show similar low free energies, and free energy alone may not be sufficient to discern the best structure prediction. Thus, looking at a group of suboptimal, low-energy structures can provide additional insight into RNA structure and function. Many approaches to sampling suboptimal structures, including base pair probabilities, Boltzman centroids, and abstract shape analysis, provide a broad view of the RNA folding landscape [6–10]. The incorporation of experimental constraints from phylogenetic analyses or chemical and enzymatic probing in addition to thermodynamic energy minimization can also help identify and define the most functionally relevant secondary structure. However, chemical probing may not necessarily define a single unique structure, and then exploring possible diverse low energy structures is necessary to understand and predict RNA structure-function relationships.

The complete enumeration of all possible RNA structures can be a useful complement to sampling approaches when testing specific hypotheses or logic conditions. A complete search of conformational space or at least a complete set of solutions for a defined region of conformational space is necessary to nullify a logical statement. For example, a combinatorially complete analysis of possible Watson-Crick helices in the Satellite Tobacco Mosaic Virus (STMV) RNA sequence demonstrated to be false the hypothesis that the 30 helices of 9 pairs observed in the STMV crystal structure contained only Watson-Crick pairs [11]. This result then led to further consideration of helices with noncanonical pairs and provided support for studies of many more possible STMV RNA structures. Sampling approaches by design do not combine suboptimal substructures [5,10,12] and thus do not provide the same insight into the region of conformational space around the free energy minima as the insights from a complete set of solutions. In this manuscript, a combinatorially complete approach is used to test whether thermodynamic free energy minimization and chemical probing can define a single, unique RNA structure.

The complete enumeration of all possible folds for an RNA sequence was first demonstrated for tRNA by Pipas and McMahon [13]. The Crumple algorithm efficiently computes all possible non-pseudoknotted structures for sequences up to approximately 60 nucleotides [14]. The number of possible structures grows exponentially with the length of the sequence, however. For longer sequences, the Wuchty algorithm combines a combinatorially complete approach with a filter for free energy minimization [15]. The Wuchty algorithm computes all possible folds for an RNA within a given window of free energy above the minimum free energy structure.

Expanding the window of free energies in the Wuchty algorithm allows for consideration of higher energy structures that may present different shapes for RNA-protein interactions and tertiary interactions with substantial free energy contributions. For example, chemical probing constraints *in vivo* indirectly account for protein and tertiary interactions, and the incorporation of these chemical probing data in RNA structure prediction often generates structures that are more accurate to the functional fold but also have less favorable energy than the predicted minimum free energy structure without chemical probing constraints [3]. This manuscript presents a parallel version of the Wuchty algorithm with additional filters for experimental data. The additional filters include: context-dependent constraints for chemical probing, optional multibranch loops, and the minimum number and length of helices. The minimum number and length of helices is a filter applied to the output of the dynamic programming algorithm. The efficient parallelization is demonstrated with several natural sequences, including tRNA, group I introns, and satellite tobacco mosaic virus RNA. These are useful improvements on a tool to generate hypotheses about RNA structure and function and provide an expanded view of the folding landscape for an RNA sequence.

## Wuchty Algorithm

We first present a summary of the Wuchty algorithm in terms of the Nussinov schema in order to provide a context for the importance of the modifications and new experimental filters we have developed. The input for the Wuchty algorithm is an RNA sequence, a database of thermodynamic parameters, a maximum free energy value for possible structures, and experimental constraints. The output is a combinatorially complete set of possible structures within the defined energy window. The first step of the Wuchty program computes a minimum free energy structure for the sequence and generates a matrix of free energy values for substructures using the Zuker algorithm [10]. The asymptotic complexity of the Wuchty algorithm is not changed by parallelization or additional experimental constraints.

**Fig 1:**
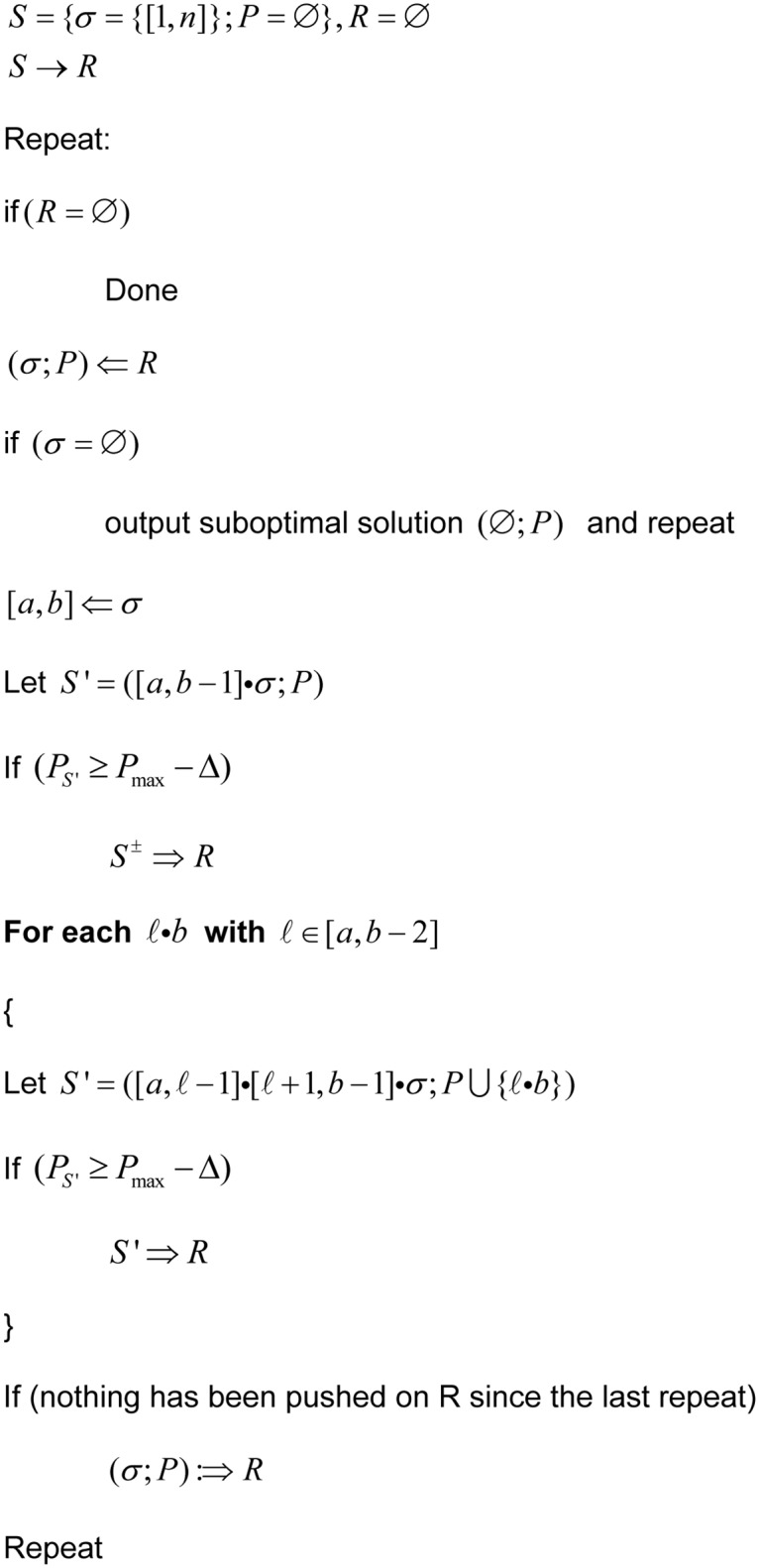
Pseudocode for the generation of suboptimal structures with the Wuchty algorithm. The pseudocode for the Wuchty algorithm is reproduced from the original description of the algorithm in reference [15]. P is a set of pairs i, j. S is a partial structure. σ is a stack. ∅ is an empty stack. R is a stack of partial structures. ℓ is the length of the sequence. Bold text highlights where new constraints were applied to the evaluation of a base pair.

In the Wuchty algorithm [15] (Fig. 1), a state is defined as a list of unevaluated intervals σ and a set of pairs P. A stack R is initialized with a state whose interval list contains only [1,n] and where P is empty, which describes a completely undetermined secondary structure whose free energy is zero. N is the number of nucleotides in the input sequence. A loop is entered in which a state S = (σ, P) is retrieved from the stack and evaluated. Evaluation consists of removing interval [i,j] from σ. A child state S′ = (σ+[i,j-1], P) is considered in which j does not pair. As pairs are added to P its free energy is updated to account for stacking and nearest-neighbor effects. Zuker’s dynamic backtrack tables [10] are used to estimate the least possible energy that can be achieved in the unevaluated intervals. If deltaG_P_S′ + deltaG_σ_S′ is below the maximum free energy threshold, then S′ is considered a viable partially determined structure and is pushed onto R for evaluation in a subsequent loop iteration; otherwise S′ is discarded. Next, we consider each potential pair (i,k) where i < = k < j. The free energy of S′ = (σ+[i,k-1]+[k+1,j-1], P+(i,k)) is likewise estimated using Zuker’s tables and thermodynamic parameters for the unevaluated intervals and stacking and nearest-neighbor effects on the determined portion P. If S′ is viable, it is pushed onto R for evaluation in a subsequent loop iteration; otherwise it is discarded. Finally, since [i,j] was removed from σ, we can add S′ = (σ, P) back onto R if we have not added any states child states this loop iteration.

**Fig 2:**
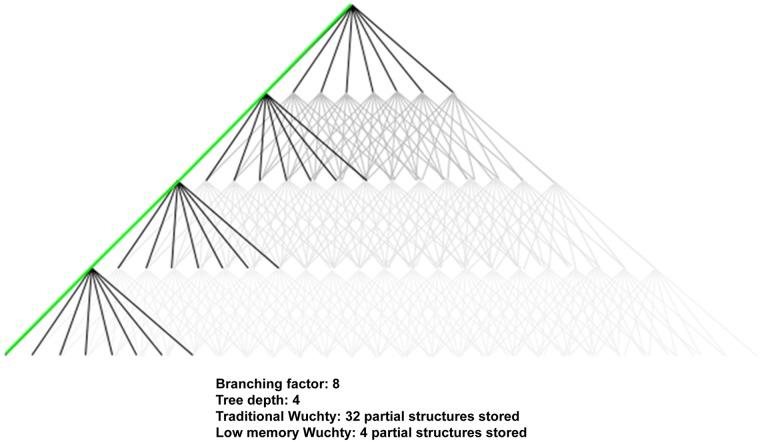
Tree Structure of Wuchty and Low Memory. The lines connect the point between a parent state and a child state and abstractly represent the tree structure of the Wuchty algorithm. The points at the bottom are leaves of the tree and represent a completely computed RNA secondary structure. The dark lines highlight the parts of the tree that are stored in memory during the computation of the first structure by the original Wuchty algorithm. The green line highlights the parts of the tree stored in memory by our ‘low memory’ modification. “Only one leaf of the calculation for the complete tree (shown in gray) is highlighted for clarity in this example.

The derivation of the S′ child state from the S parent state suggests a convenient tree description in which the completely undetermined root is the ancestor of all states, and leaves are completely determined secondary structures. The states in the stack R represent the unevaluated fringe of the tree. An intermediate node represents a partially determined state, the set of whose determination is a superset of its ancestors’ and a subset of its descendants’ determinations. The branching factor of the tree is proportional to the sequence length. Fig. 2 shows an example abstract tree diagram with a depth of 4 and branching factor of 8.

The original implementation of the Wuchty algorithm for suboptimally folding secondary structures requires working memory proportional to the length of the sequence and the size of the accepted energy window. This has made memory the limiting factor for producing large sets of suboptimal structures. The Wuchty algorithm is a method for exploring a tree of possible secondary structures. Each leaf of the tree represents a valid secondary structure. Each node on the tree represents a partially-completed structure, which could be completed in many ways—each possible refinement of a partially completed structure is represented by a branch stemming from that node. The most direct translation of the Wuchty algorithm results in an implementation which creates and stores all the immediate children of a node in a stack of nodes needing refinement. The process is then repeated for the top node in the stack, until the top node is a completed structure, ready to be saved to disk. This process requires that, before the first complete secondary structure can be saved permanently to disk, working memory must contain a number of states equal to the length of the sequence time the branching factor of the tree. The branching factor of the tree is linear with respect to the length of the sequence, resulting in >O(N^2^) memory usage. For long sequences, this becomes unmanageable, even with considerable computing resources.

## Computational Improvements and Results

### Wuchty suboptimal folding with lower memory use

A variation of the Wuchty approach is to store only a single branch at each node, along with enough information to know what (as yet unbuilt) branch should be explored next. This amounts to storing the current indices of iteration for each loop executed during the production of child-states. Only when the current child-branch has been completely explored should the next child-branch be created, by continuing the examination of the parent from exactly where that examination left off previously. Only one branch is stored per node at any moment, and so the memory needed before the first solution can be saved to disk is linear with respect to the sequence length. This results in reasonable working memory usage even for long sequences, making time the limiting factor in suboptimal folding, instead of memory. This approach works well in serial computations, but to truly take advantage of this approach in parallel would require a fresh implementation. Thus, the parallelization of the Wuchty code proceeded with the original Vienna group implementation of the Wuchty algorithm [15,16].

### Parallelization

**Fig 3:**
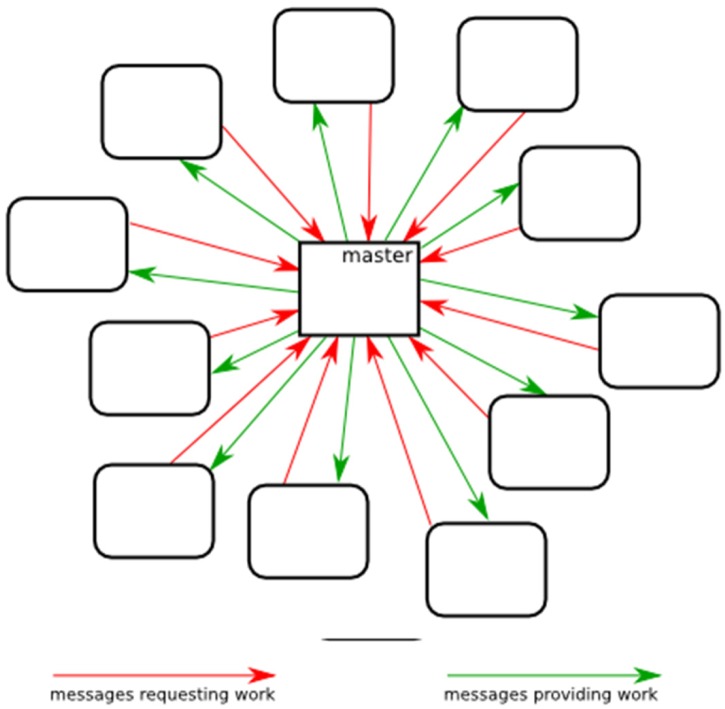
Parallelization Scheme. The parallelization scheme shows direct communication between each core and the master core.

**Fig 4:**
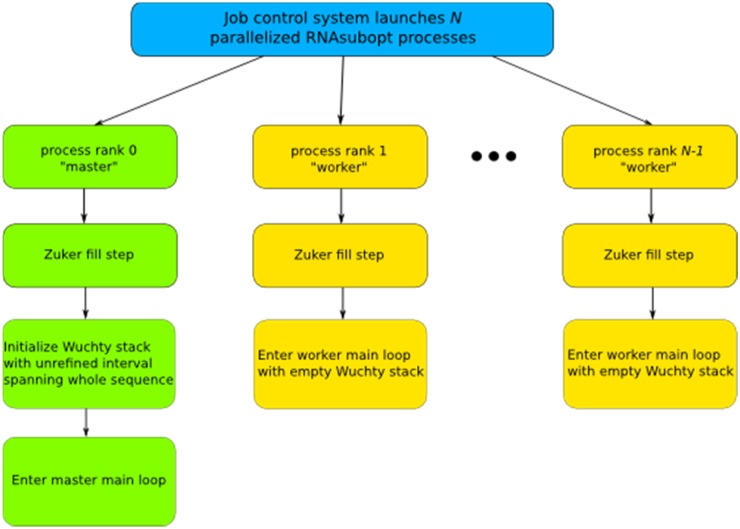
Flowchart diagram for initialization of parallelized RNA subopt.

**Fig 5:**
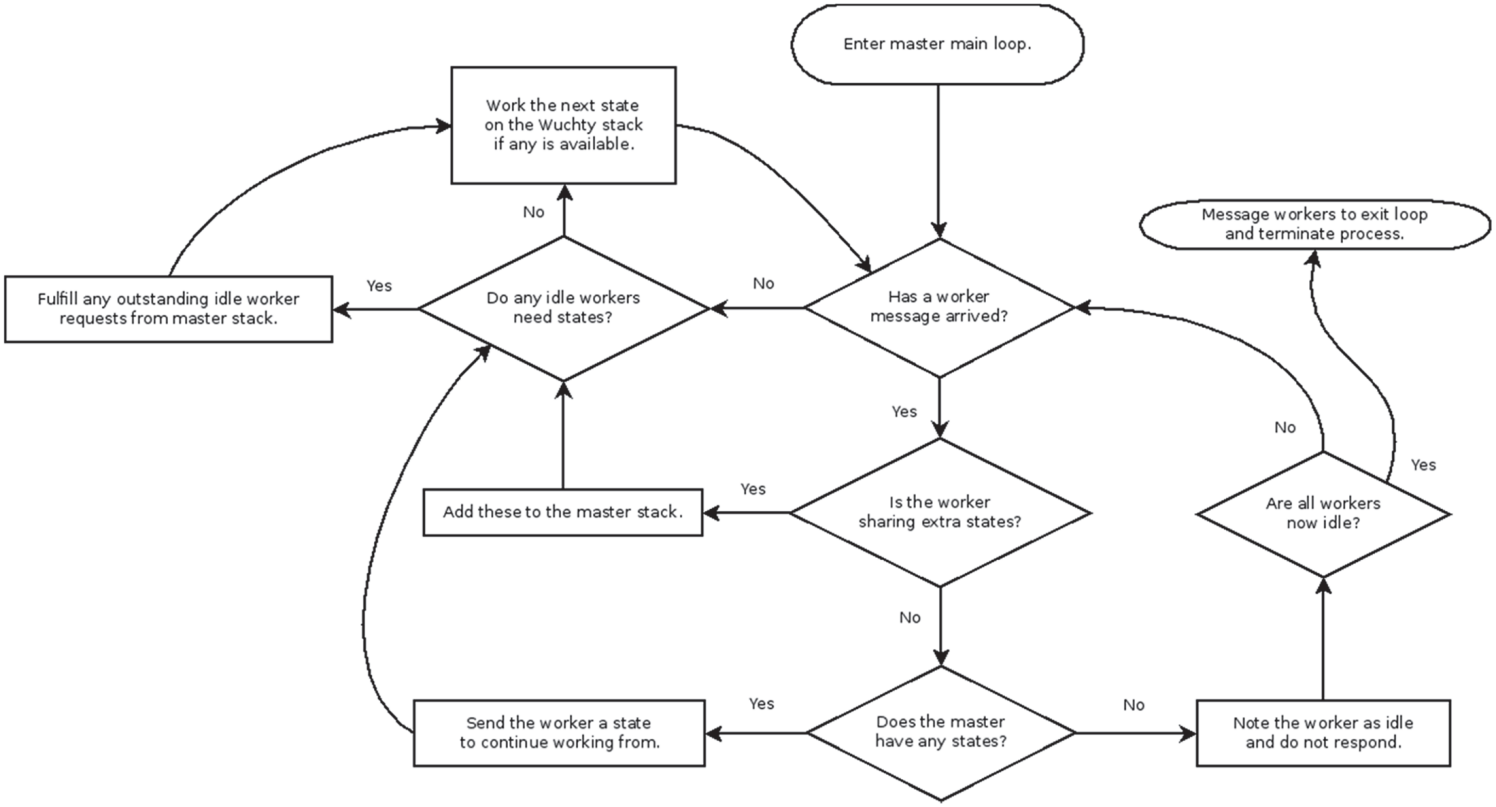
Flowchart diagram for master loop.

**Fig 6:**
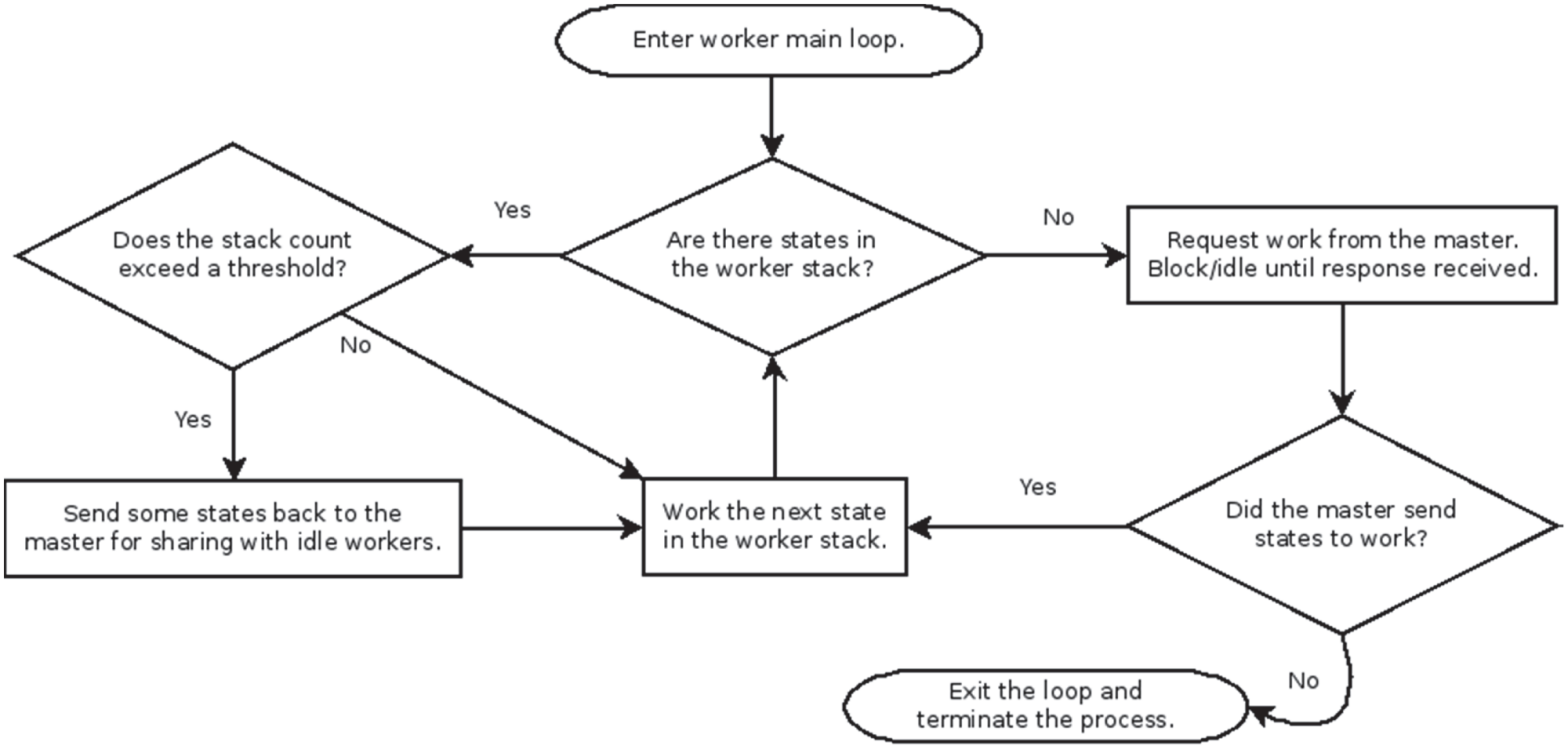
Flowchart diagram for worker loop.

In a serial run, the Vienna implementation starts by computing the Zuker energy arrays and then initializing the refinement stack. The next step is entering the main loop where one state is extracted from the stack at a time, and its child states are determined and pushed back onto the stack for evaluation in subsequent loop iterations. A parent state is computed before a child state can be computed. A parent state’s children may themselves be worked in arbitrary order relative to each other, opening the possibility of efficient parallelization. In our parallel adaptation (Figs. 3–6), a P-process run consists of 1 master process and P-1 worker processes that each begin the same way as a serial scheme by computing its own identical copies of the Zuker arrays according to the run parameters, which simplifies subsequent communications. The Zuker fill step requires a negligible amount of time that does not benefit from parallelization. The parallel scheme begins to differ from the serial scheme with the initialization of the refinement stack. The master process initializes its stack with the completely unrefined root node state containing a single unevaluated interval spanning the length of the sequence, and the worker processes initialize their stack as empty, i.e. containing no refinements. The master process enters its main loop (Fig. 5) and the worker processes enter their main loops (Fig. 6). Worker processes communicate with the master via message passing interface (MPI). A worker can send two types of messages to the master. The first indicates that the worker’s stack is empty, and so it is idle and needs work. The master can respond to this message in one of two ways. The master can cause the worker to resume from an idle state by sending back a state from its own stack for the worker to populate its stack and begin working in the next loop iteration, or the master can send a message indicating that no work remains to be done and the worker should terminate. Thus, when the worker loop is initially entered, its empty stack prompts a message to the master indicating a need for work. The master loop begins by answering any worker messages or, if no worker messages have arrived, working the next state in its stack itself to create more child states which can be shared with idle workers.

As workers’ states beget new child states, the states are added to the workers’ own stacks. In a serial run, the stack grows arbitrarily large with child states. However, any given state’s child count may be as large as the size of the current interval being evaluated or as small as zero when none of its children viably accommodate the run parameters. The number of child states refined from a parent under given parameters can only be determined when the parent state is evaluated. It is not possible to ensure that when each of several workers are assigned states to work, they will all take exactly the same amount of time and stay busy for the duration of the run. Some states yield billions of descendants under certain run parameters, and some yield comparatively few. This motivates the second message a worker sends to the master, which indicates that the number of states on the worker’s stack has exceeded a configurable threshold. The worker sends with this message an excess of states removed from its stack, which the master receives and adds to its own stack for distribution among workers that become idle. Sharing a common refinement stack among multiple processes in this way yields a complete and non-duplicating exploration of the structure space due to the independent nature of Wuchty state refinements. Worker starvation and serial degeneration is thus avoided, and maximal occupancy of worker processes is enforced

**Fig 7:**
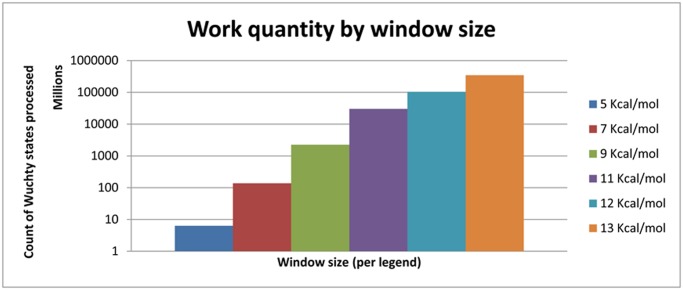
Work Quantity by Window Size. The number of states within a given energy window increases exponentialy with window size. The sequence used is 247 nucleotides from the *Tetrahymena thermophila* group I intron crystal structure PDB ID IX8W [33]. The computations use the revised version of no lonely pairs constraint, 2004 thermodynamic parameters [3], and the Boomer supercomputer. Color legend: 5 kcal/mol, gray diamonds; 7 kcal/mol, red squares; 9 kcal/mol, green triangles; 11 kcal/mol, purple x’s; 12 kcal/mol blue asterisks; 13 kcal/mol, orange circles.

**Fig 8:**
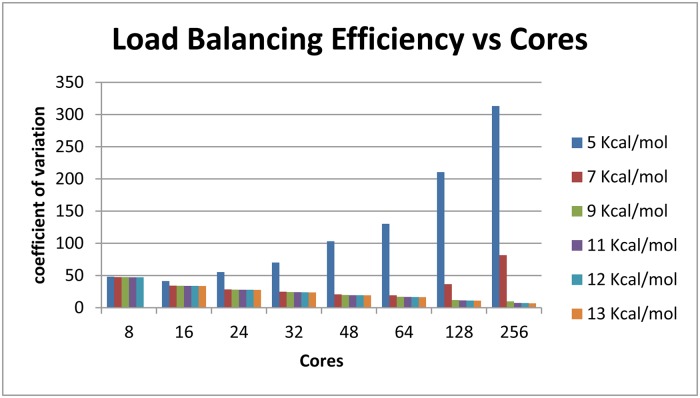
Load Balancing Efficiency. The load balancing efficiently depends on the amount of work and the number of cores. The computational input and parameters and color legend are the same as fig. 7.

**Fig 9:**
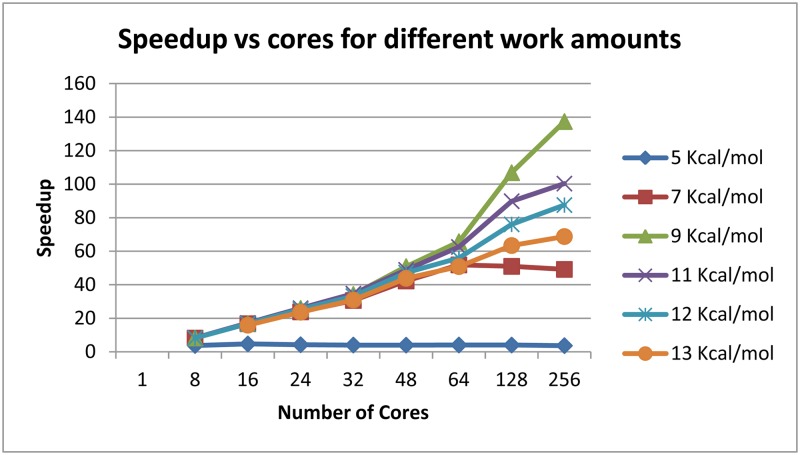
Speedup for Parallelization. Speed up is the ratio of real computation time in serial to real computation time in parallel. The computational input and parameters and color legend are the same as for figs. 7 and 8.

Efficient load balancing can be done without actually calculating how much work a branch represents. Work increases exponentially with window size as indicated in Fig. 7. Load balancing efficiency tends to increase with available work because the likelihood that a worker node remains idle requesting work is significantly decreased (Fig. 8). Actual speedup, the time benefit to using parallelization over serial computation, increases with total work up to a certain point and demonstrates diminishing returns after some point. This effect is sequence dependent. For *Tetrahymena thermophila* group I intron, a 64-process run shows nearly linear speedup for non-trivial energy windows. Linear speedup is the best possible result for this type of parallelization. With greater than 64 processes, it is still effective to use more cores as available, but the returns are diminishing. A 9 kcal/mol *T*. *thermophila* group I intron run with work quantity on the order of billions of units demonstrates a speedup of approximately 140x with 256 processes, about half the speedup-per-core of the 64 process trial (Fig. 9).

The work quantity by window size graph (Fig. 7) demonstrates that number of states to be analyzed in execution of the steps of the Wuchty algorithm increases exponentially as the thermodynamic window size increases linearly. Keeping in mind the Wuchty tree, states are non-leaf nodes and solutions are leaf nodes. When no thermodynamics are considered (i.e. an infinite thermodynamic window), all combinatorially possible solutions are enumerated. The quantity of solutions can be estimated at 1.8^N^, where N is the sequence length [17]. For smaller values of N it is possible to enumerate all possible solutions and thereby arrive at an exact solution count. All values are sequence-specific, i.e. two different 14mers will likely have different exact solution counts on the same order of magnitude. For the 14mer sequence 5’GCUCUAAAAGAGAG, there are 119 combinatorially possible solutions. The results of the 14mer can be checked manually. The sequence of the 14mer is designed to contain one example of each possible case of context-dependent chemical probing (see below). For a concatenation of two or three of these 14mer sequences, there are 124,926 and 197,316,085 solutions respectively for the 28mer and 42mer. The number of leaf nodes, or solutions, is on the order of the number of non-leaf nodes, or refinements in the context of the original Wuchty algorithm. Fig. 9 demonstrates the state counts, i.e. the total states processed across all runs, at various energy windows for the 247-nucleotide *Tetrahymena* sequence. It is clear that the amount of work increases exponentially as the energy window increases. With the 1.8^N^ estimate, there may be as many as 1.12x10^63^ combinatorially feasible solutions to the 247-nt *Tetrahymena* sequence.

The load balancing efficiency versus cores graph (Fig. 8) demonstrates that as the amount of work to be done increases, the work becomes more evenly balanced across the processor cores participating in the run. The amount of work to be done is measured by the state counts described in the work quantity by window size. A degenerate case has minimal work, many idle workers, and large communicative costs. In the degenerate case of a 5 kcal/mol energy window with fewer than 10 million states, adding cores beyond 16 actually decreases the efficiency of load balancing because the ratio of each core’s time spent communicating versus time spent working through states actually increases. The same phenomenon is observed in a 7 kcal/mol window with just over 100 million states—load balancing efficiency increases up to 64 cores, while the efficiency decreases when utilizing 128 cores. Both the 5 kcal/mol and 7 kcal/mol runs can be completed in serial in a reasonable amount of time. At the other extreme, the 13 kcal/mol run represents hundreds of billions of states, and each addition of processor cores increases the efficiency of load balancing.

The speedup versus cores for different work amounts demonstrates the actual time-value of adding processor cores to a given parallel Wuchty run (Fig. 9). At a 5 kcal/mol window, fewer than 10 million states do not get worked more quickly by 256 cores than they do by 8 cores. Runs with windows of 9 kcal/mol and greater continued to see increases in speedup as cores were added up to 256.

### No Lonely Pairs Modification

**Fig 10:**
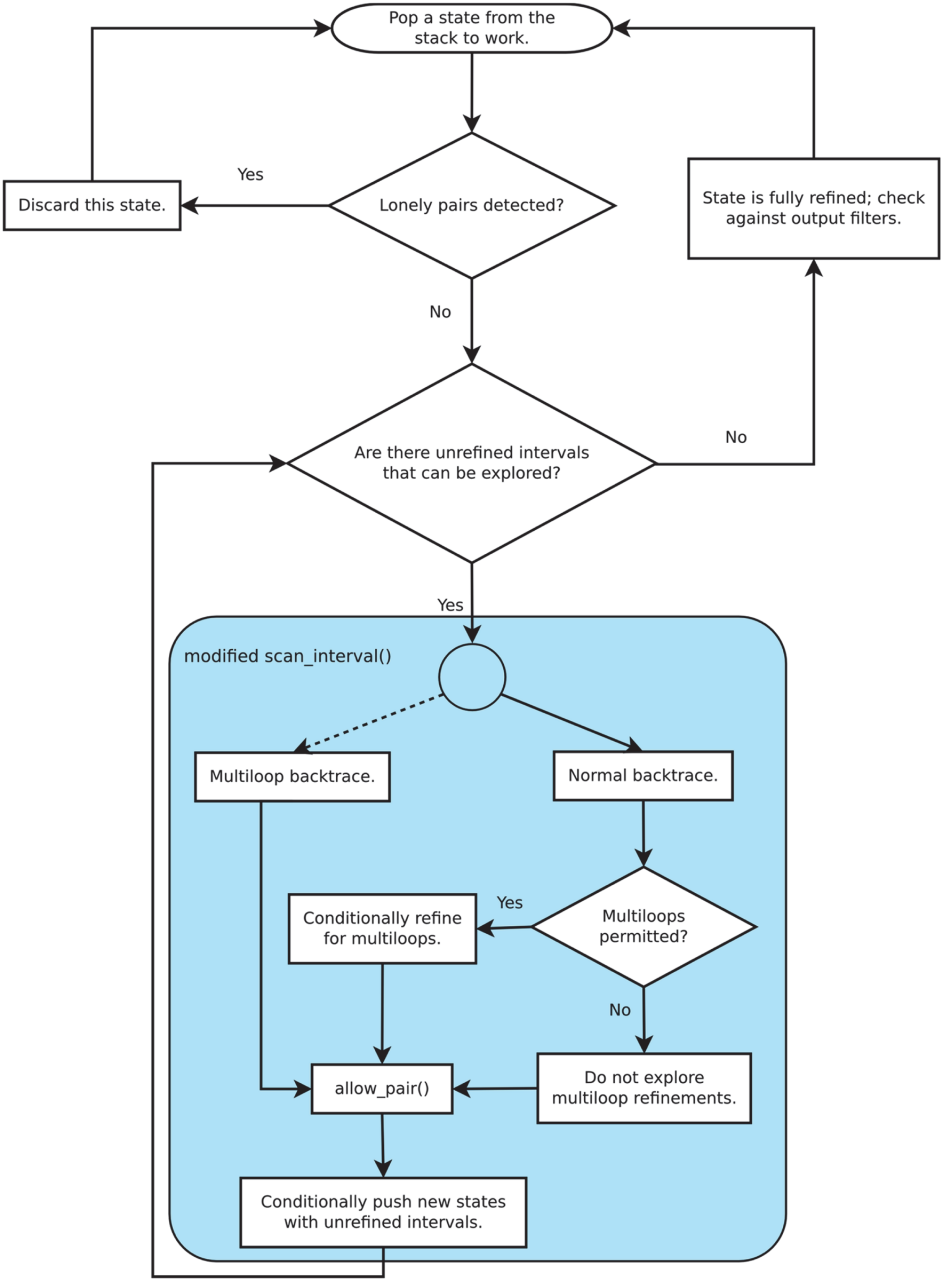
Flowchart for scan interval. The assessment of lonely pairs, the optional multibranch loop, and the allow pair function are new modifications to the original scan interval code in the Vienna Package.

The no lonely pairs option in the Vienna Wuchty algorithm uses a large energetic penalty to prevent generating structures with isolated single pairs that do not stack on a neighboring pair. The traditional energy penalty approach works well in many contexts [18]. However, if the window size being explored happens to be chosen larger than the energy penalty, then structures with lonely pairs may occur in the output. Thus, an alternate no lonely pair strategy was developed. The original no LP condition is not evaluated, and no energy penalty is applied. Each partial structure is evaluated after each iteration of the algorithm. When a lonely pair forms in a partial structure during an iteration and no interval remains in the refinement that could permit the lonely pair to have a neighbor, the branch is pruned by simply discarding it rather than pushing it onto the refinement stack (Fig. 10).

### Additional Experimental Filters

Pruning occurs when a decision is made to leave a fringe node unexplored. The original Wuchty algorithm prunes branches in which thermodynamic stacking and nearest-neighbor effects are not sufficient to bring free energy below the configured threshold. There are several ways to incorporate additional experimental filters that further prune branches of the tree at the initial consideration of whether a base can pair, during the growth of a branch, and at the leaves of the tree. The earliest time to prune a branch is the decision of whether a base can pair. The pairing rules for Watson-Crick and GU pairs are the most basic example of this kind of constraint. The original Wuchty algorithm also includes hard constraints for single-stranded nucleotides that are identified by S1 nuclease or chemical probing, and these nucleotides are marked with an”X” as non-pairing. If modified nucleotides that cannot form Watson-Crick pairs are known in the sequence, then these nucleotides can also be marked as nonpairing with an “X.” The original Wuchty algorithm also includes constraints that prune all conformations that are incompatible with a constrained pair between position I and j. Covariation data from phylogenetic comparisons can constrain two nucleotides to form a pair. In the context of an algorithm that excludes pseudoknotted pairs, the covariation constraint restricts the pairing possibilities for nucleotides between the two covarying nucleotides, ie the nucleotides in between may only form pairs with each other. Thus, pairing constraints have a much larger effect on reducing conformational space than single strand constraints, as was previously demonstrated with the Crumple algorithm [14].

During the computations that explore branches of the tree, the maximum base pair distance constraint keeps pairing within an upper bound. Nucleotides at a farther distance and those subsequent branches are not considered at all. This constraint can be a useful approximation for folding domains within an RNA sequence or incorporating models of cotranscriptional folding, which implies only local pairing, as demonstrated by the COFOLD program [19], although there is some debate about the use of this constraint [20]. The computations presented in this manuscript do not use this maximum base pair distance constraint. We instead developed a user option for multibranch loops that does not impose any constraints on how far away pairing nucleotides are in the sequence.

A new tool introduced in this version of the Wuchty algorithm makes the formation of multibranch loops a user option by introducing modifications in the scan_interval step in the Wuchty code (Fig. 10). Multibranch loops form when long range pairs form at a junction with more than two helices and create nested helices. Wuchty implements multiloops first in the Zuker fill step by assigning a favorable energy to the pairs that would close multiloops, and then in the suboptimal backtrack step by evaluating the stacking relationships within intervals enclosed by such pairs with favorable energies. Note that the pairs closing a loop can be assigned a favorable energy, although multibranch loops have unfavorable energy parameters in the 2004 Turner rules [3]. We disable multiloops in the fill step by not granting the favorable energies to pairs that might close multiloops and then in the backtrack step by simply not exploring multiple hairpin loops within a single hairpin loop. This feature is also useful for generating possible structures that would be consistent with cotranscriptional folding, which implies only local pairing in a series of hairpins. This user option demonstrates the effects of long-range pairing in multibranch loops on the energies and diversity of possible RNA structures.

Context-dependent constraints for chemical probing can also prune branches of the tree. Nucleotides in Watson-Crick pairs at the ends of helices, adjacent to internal loops and bulges, or adjacent to GU pairs can also be chemically modified by reagents such as dimethyl sulfate. Nucleotides in Watson-Crick pairs in the middle of two other Watson-Crick pairs cannot be chemically modified. Unlike hard constraints that force a nucleotide to be unpaired always, this is a context-dependent constraint that depends on the adjacent nucleotide pairing. Allow_pair is a software filter that prunes branches based on nucleotide solvent accessibility, greatly reducing the number of intermediate states and leaves (Fig. 10). Allow_pair considers the pairing of adjacent nucleotides to effect a soft, context-dependent constraint, as originally described in Mathews et al. [3]. The implementation of allow_pair differs slightly from the implementation of soft constraints in the new version of the Vienna software packaging [21] but achieves a similar result in allowing context-dependent chemical probing constraints.

The completely determined secondary structures in the leaves of the tree can be examined by software filters such as helix filter, which counts the number and size of helices and does not print those that do not meet user-defined criteria. Helices are formed during the backtrack step, and the final number and sizes cannot be known until the state is fully refined and the corresponding structure completely generated. Thus, this filter is applied at the last stage of generating an output structure. Attempts to prune earlier in the tree based on the minimum number of remaining unassigned nucleotides necessary to form the minimum number and length of helices had a negligible effect on computations. The rules for counting helices are adapted from the Helix_Find algorithm [11]. This constraint is useful for problems such as Satellite Tobacco Mosaic Virus (STMV) RNA and MS2 bacteriophage RNA, where the minimum number and lengths of helices encapsidated in the virus particle are known from crystallography or cryoelectron microscopy [22–24]. RNA viruses such as canine parvovirus, Flock House virus, mouse minute virus, pariacoto virus, Q-beta bacteriophage, Hepatitis B pregenomic RNA, and cowpea chlorotic mottle virus (CCMV) also show electron density for ordered RNA helices within the viral capsid, which suggests that structured encapsidated nucleic acid occurs throughout a wide range of viruses [25]. Thus, a helix filter will be useful for modelling other encapsidated viral genomes.

### Chemical and Enzymatic Probing Constraints Reduce Conformational Space but Do Not Define a Single Structure

**Fig 11:**
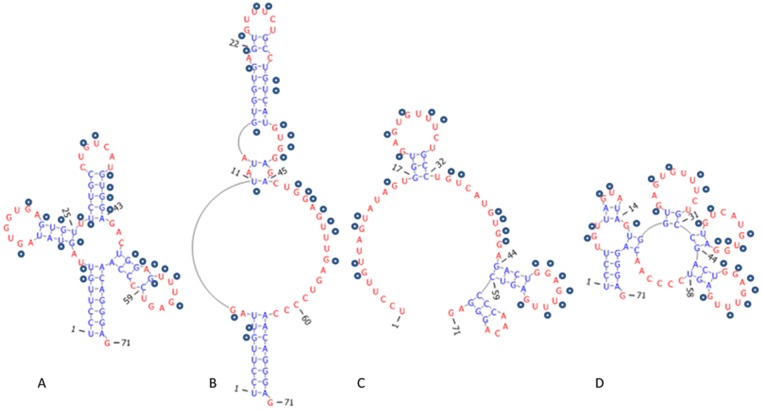
Alternate folds for tRNA Asp 7. (A) RNA fold for tRNA Asp 7 sequence based on phylogenetic data. Circles represent experimentally measured single strand S1 nuclease hits [26]. (B) Alternate functional RNA fold proposed in [26]. The large asymmetric loop leaves nucleotides available for pairing with an Alu sequence. (C, D) The minimum free energy fold and an alternate fold from computations with S1 nuclease data as a hard single-strand constraint.

The human tRNA Asp 7 sequence has two functional conformations, the traditional cloverleaf structure and an alternative structure that binds Alu domains [26] (Fig. 11). This tRNA sequence provides an excellent test case for exploring the effects of enzymatic probing constraints on the possible conformational space. Table 1 shows the results of several parallel Wuchty runs for the human tRNA Asp7 sequence with no constraints, hard single strand constraints from S1 and T1 enzymatic probing, and context-dependent chemical probing constraints using the allow-pair option. The base pair distance is a measure of how many nucleotides are differently paired in two RNA structures and computed using the rna_dist function in the Vienna Suite [16]. (Note: The base pair distance that describes the number of differently paired nucleotides in two RNA structures is distinct from the constraint “maximum base pair distance” when folding a single RNA that is described earlier in the text.) The hard single strand constraints reduce the possible conformational space much more as demonstrated by a higher minimum free energy value, fewer structures in a larger window size, a smaller maximum base pair distance value, and a smaller average value for base pair distance. However, the constraints do not yet define a single conformation (Fig. 11). In contrast, the context-dependent single strand constraint reduce the number of possible structures in the same window size (19.37 kcal/mol) but do not significantly affect the minimum free energy value, the window size, or the diversity of possible structures as indicated by the similar maximum and average base pair distance values. 6 nucleotides are hit by both the single strand S1 nuclease and the double strand V1 nuclease, which occurs when the V1 nuclease sometimes recognizes and cleaves a stacked nucleotide that is not paired. This type of ambiguity is difficult to resolve in generating appropriate constraint lists. The use of context-dependent chemical probing constraints that allow pairing at the ends of helices can resolve some conflicting S1 and V1 constraints. Thus, the enzymatic probing constraints reduce the possible structures for the RNA sequence but have different effects on changing the shape of the conformational landscape and do not unambiguously define a single structure.

**Table 1:** Effect of Single Strand Constraints on the Conformation Space of tRNA Asp 7.

Constraints	MFE (kcal/mol)	Window Size	Number of Structures	Maximum Value Base Pair Distance	Average Value Base Pair Distance
None	-19.37	19.37	337,145,797	48	23.45
23 hard single strand	-8.94	35.76	949,437,420	28	17.76
23 soft single strand	-19.37	19.37	139,643,411	48	24.74

The sequence for human tRNA Asp 7 [26] was folded using the Wuchty algorithm with the no lonely slow option and 2004 thermodynamic parameters and the largest possible window size in a 48-hour computing time. Hard single strand constraints require the nucleotide to be unpaired. Context-dependent single strand constraints allow nucleotide pairing at the end of helix, adjacent to loops, and adjacent to GU pairs. The maximum and average base pair distance values are calculated using the rna_distance function in the Vienna Package. The base pair distance is a count of how many base pairs exist in one structure and not another when comparing two RNA secondary structures. All computed structures were compared to the MFE structure in the rna distance calculations. Note: this use of “base pair distance” is not to be confused with the constraint (not used in these RNA structure predictions) that limits how far away a nucleotide can pair with another nucleotide within a single structure.

### STMV RNA

The parallel version of the Wuchty algorithm with chemical probing constraints and helix filters is able to explore a larger window of free energy for longer sequences, such as STMV RNA that has 1,058 nucleotides. These computations on a larger free energy window provide insight on the shape, density, and diversity of the folding funnel for an RNA sequence. For example, previous computations with the STMV RNA sequence (accession number NC_003796.1) using the Wuchty algorithm as implemented in RNAStructure were able to explore only a 1 kcal/mol energy window due to memory constraints [5]. The STMV RNA sequence can fold into 42,768 different structures within 1 kcal/mol. This set of structures includes two structures with 560 nucleotides differently paired that have free energies different by only 0.2 kcal/mol. In computations using the STMV RNA sequence and the modifications presented in this paper for the Wuchty algorithm, a 5 kcal/mol energy window was able to be explored. The STMV RNA computations use 161 single strand constraints, the energy-independent no lonely pairs option, the 2004 thermodynamic parameter set, a helix filter for 30 helices of 9 pairs with up to 3 mismatches per helix, and optional multibranch loops [11]. The formation of multibranch loops is more consistent with models of RNA folding by free energy minimization while the formation of a series of stem loop hairpins is more consistent with models of cotranscriptional folding and virus assembly [11,23,27]. 4,862,164,626 structures without the multibranch loop option were generated in 9,438 s (2.62 h) using 256 cores. 1,159,185,578 structures with the multibranch loop option were generated in 49,485 s (13.74 h) using 256 cores. The maximum base pair distance in this set of structures was 306 nucleotides paired differently, which suggests a wide diversity in the structures. The minimum free energy for structures computed with the multibranch loop option is-341.6 kcal/mol, while the minimum free energy for structures computed without the multibranch loop option is-289.2 kcal/mol, indicating that structures with very different conformations and energetic stabilities can satisfy the same set of constraints. Thus, over 6 billion structures exist in a greater than 50 kcal/mol energy span that all satisfy the chemical probing and crystallography constraints for encapsidated STMV RNA. The conformational space for encapsidated STMV RNA therefore resembles a wide, flat basin rather than a folding funnel that converges to a single unique structure.

## Discussion

The new modifications to the Wuchty algorithm presented here, including parallelization, energy-independent lonely pair constraints, context-dependent chemical probing constraints, helix filters, and optional multibranch loops, provide useful tools for exploring the landscape of RNA folding. The parallelization scheme takes advantage of modern computing resources to expand the energy range that can be explored for longer RNA sequences. The parallelization is efficient and increases in efficiency of load balancing as the workload increases. As a wider energy range is explored, the importance of experimental constraints also grows. The new implementation for lonely pair constraints does not depend on energy penalties and is most useful in cases where combinatorially complete sets are computed that include high energy structures. The context-dependent implementation of chemical probing constraints rather than hard single strand constraints allows more possible structures to be consistent with chemical probing data. The use of these context-dependent constraints resolves potential conflicts between chemical probing constraints, which identify unpaired nucleotides as well as nucleotides paired at the ends of helices, and constraints from V1 nuclease, which identifies double stranded nucleotides and also sometimes stacked but unpaired nucleotides.

The helix filters and multibranch loop option are constraints on the global RNA secondary structure rather than nucleotide specific constraints. To our knowledge, these constraints for optional multibranch loops and helix filters are not yet available in any other free energy minimization tool for RNA structure prediction of suboptimal folds. The Unafold package allows optional multibranch loops in partition function calculations for a single minimum free energy structure [28]. The helix filters identify structures with a specified number of helices of a user-defined length and mismatch tolerance. These filters incorporate data from crystallography or cryo-electron microscopy that is especially useful for studying the folding landscape of encapsidated viral RNA. The option to disallow multibranch loops is useful for generating structures consistent with models of cotranscriptional folding and viral assembly. Cotranscriptional folding and viral assembly posits that the RNA folds in a series of local hairpins and then viral capsid proteins bind these hairpins during transcription and begin virus assembly [27]. In this kinetic model, long-range interactions such as multibranch loops are less likely to determine RNA folding. The no multibranch loop constraint forces a series of hairpins to form without limiting the distance allowed for nucleotide pairing. This constraint changes the minimum free energy structure significantly and then allows a different region of the folding landscape to be computed using the Wuchty algorithm.

### Experimental constraints define a region of conformational space rather than a single structure

Incorporating diverse experimental constraints defines the possible regions of conformational space for an RNA sequence. However, chemical probing does not necessarily identify unambiguously a single unique secondary structure as demonstrated by the tRNA Asp sequence [26] and the ensemble of structures for encapsidated STMV RNA and MS2 RNA [11,23]. Thermodynamic parameters for RNA motifs remain one of the most powerful constraints for defining possible RNA structures [3]. However, a single minimum free energy structure for an RNA sequence of 1,000 nucleotides such as STMV RNA has a very low probability of occurring [29] and thus methods to compute centroid structures [30], base pairing probabilities [9], shape analysis [6], and suboptimal structures [10] are necessary to identify functional RNA conformations.

The Wuchty algorithm [15] produces a combinatorially complete set of structures within a defined range of thermodynamic stabilities. A combinatorially complete approach is useful for defining the landscape of RNA conformational space, even if only for a local region. The modifications presented in this work expand the region that can be defined and computed completely. Although combinatorial approaches may produce too many structures to be of practical use for many biological applications, combinatorial approaches can be a useful tool for theoretical applications and logically eliminating some possible solutions. For example, a combinatorial approach in the *Helix Find and Combine* program eliminated the possibility of any structure with thirty helices of nine perfect pairs for the STMV RNA sequence, even when multibranch loops and pseudoknots were considered [11]. Thus helices with mismatches must be present in possible solutions for encapsidated STMV RNA. In this work, using the Wuchty algorithm to study STMV RNA produced billions of diverse structures consistent with experimental data within a 5 kcal/mol energy window. This result does not eliminate the possibilities of solutions with or without multibranch loops but does make a solution containing only a single structure highly improbable.

Our previous model for encapsidated STMV RNA presented an ensemble of helices, of which any non-overlapping combination of thirty helices would constitute a valid solution [11]. This approach focused on experimental constraints from crystallography and chemical probing and does not require thermodynamic parameters. The previous ensemble and highest scoring secondary structure was intentionally underpredicted in order to facilitate three-dimensional modeling of the STMV particle. The results in this work are complementary to the previous results with Crumple, Sliding Windows, and Assembly [11,14]. Both approaches show that multiple solutions are possible for folding encapsidated STMV RNA. The modified Wuchty algorithm using chemical probing and crystallography constraints generated more possible solutions without multibranch loops than with multibranch loops. However, the solutions containing multibranch loops have a lower free energy than solutions without multibranch loops. The difference in free energy for the structures with and without multibranch loops is approximately 50 kcal/mol, which is within a range that RNA tertiary interactions or RNA-protein interactions, such as the two coat proteins binding a nine-pair RNA on each of 30 two-fold axes in STMV particles [22], could be an equal or greater contribution to the overall thermodynamic free energy. Thus, although these results do not distinguish between the kinetic model for cotranscriptional RNA folding and virus assembly versus a free energy minimization model, the results more clearly define the differences between the two models as well as the overall landscape of possible conformations for encapsidated STMV RNA.

## Conclusion

This paper presents new modifications to include experimental constraints in the Wuchty algorithm that facilitate defining the possible secondary structures for an RNA sequence. The constraints for the number and length of helices and options for multibranch loops are global constraints that facilitate generating models for encapsidated viral RNA. The parallelization of the Wuchty algorithm enables a larger free energy window to be considered and a larger region of the RNA folding landscape to be defined. The results provide insight into the density and diversity of structures near a free energy minimum. Some RNA folding landscapes may resemble a wide basin, either rugged or smooth, rather than a steep folding funnel that converges to a single structure. Future efforts to identify constraints from tertiary and quaternary interactions may further reduce conformational space for encapsidated viral RNA. The computations on STMV and tRNA sequences demonstrate that chemical probing does not necessarily define a single, unique structure. The results of the Wuchty computations on STMV RNA provide additional data to evaluate models based on free energy minimization and models of cotranscriptional RNA folding and virus assembly.

### Computational Methods

The source code in C for the Wuchty algorithm from the Vienna RNA websuite 1.8.3 was the starting point for these studies [16]. The new release of Vienna 2.0 software did not change the Wuchty algorithm source code [31]. All software used in this manuscript and instructions for use are freely available at http://adenosine.chem.ou.edu/software.html and http://dx.doi.org/10.6084/m9.figshare.1276050. The programs run in conjunction with the GNU autotools build system and mercurial. This software is not supported by Windows. Tools to evaluate the differences between two RNA secondary structures and the free energy of an RNA structure, the functions rna_dist and rna_eval, respectively, are also available at http://www.tbi.univie.ac.at/RNA/ in the Vienna websuite [16]. The 2004 thermodynamic parameters from the Turner lab are used to evaluate the free energy of an RNA secondary structure [3].

Computations and programming were done on an Athlon +6400 computer at 3.2 GHz with 4 GB RAM. Computations in parallel were done using MPI [32] on the Sooner supercomputer, a Intel xeon64 quad core Linux cluster, or Boomer, a 6,992-core cluster with the Intel Xeon processor E5 family and QLogic InifiniBand adapters, which are available through the OU Supercomputing Center for Education and Research.

## References

[1] Zuker M (1989) On finding all suboptimal foldings of an RNA molecule. Science 244: 48–52. 2468181 10.1126/science.2468181

[2] Hofacker IL (2014) Energy-directed RNA structure prediction. Methods Mol Biol 1097: 71–84. 10.1007/978-1-62703-709-9_4 24639155

[3] Mathews DH, Disney MD, Childs JL, Schroeder SJ, Zuker M, et al (2004) Incorporating chemical modification constraints into a dynamic programming algorithm for prediction of RNA secondary structure. Proc Natl Acad Sci USA 101: 7287–7292. 15123812 10.1073/pnas.0401799101 PMC409911

[4] Turner DH, Mathews DH (2009) NNDB: the nearest neighbor parameter database for predicting stability of nucleic acid secondary structure. Nucleic Acids Res 38: D280–D282. 10.1093/nar/gkp892 19880381 PMC2808915

[5] Schroeder SJ (2009) Advances in RNA structure prediction from sequence: new tools for generating hypotheses about viral RNA structure-function relationships. J Virol 83: 6326–6334. 10.1128/JVI.00251-09 19369331 PMC2698544

[6] Janssen S, Giegerich R (2014) Abstract shape analysis of RNA. Methods Mol Biol 1097: 215–245. 10.1007/978-1-62703-709-9_11 24639162

[7] Giegerich R, Voss B, Rehmsmeier M (2004) Abstract shapes of RNA. Nucleic Acids Res 32: 4843–4851. 15371549 10.1093/nar/gkh779 PMC519098

[8] Ding Y, Lawrence CE (2001) Statistical Prediction of Single-Stranded Regions in RNA Secondary Structure and Application to Predicting Effective Antisense Target Sites and Beyond. Nucl Acids Res 29: 1034–1046. 11222752 10.1093/nar/29.5.1034 PMC29728

[9] McCaskill J (1990) The equilibrium partition function and base pair binding probabilities for RNA secondary structure. Biopolymers 29: 1105–1119. 1695107 10.1002/bip.360290621

[10] Zuker M, Stiegler P (1981) Optimal computer folding of large RNA sequences using thermodynamics and auxiliary information. Nucl Acids Res 9: 133–148. 6163133 10.1093/nar/9.1.133 PMC326673

[11] Schroeder SJ, Stone JW, Bleckley S, Gibbons T, Mathews DM (2011) Ensemble of Secondary Structures for Encapsidated Satellite Tobacco Mosaic Virus RNA Consistent with Chemical Probing and Crystallography Constraints. Biophys J 101: 167–175. 10.1016/j.bpj.2011.05.053 21723827 PMC3127170

[12] Mathews DH (2006) Revolutions in RNA Secondary Structure Prediction. J Mol Biol 359: 526–532. 16500677 10.1016/j.jmb.2006.01.067

[13] Pipas J, McMahon J (1975) Methods for Predicting RNA Secondary Structure. Proc Natl Acad Sci USA 72: 2017–2021. 1056009 10.1073/pnas.72.6.2017 PMC432683

[14] Bleckley S, Stone JW, Schroeder SJ (2012) Crumple: A Method for Compelte Enumeration of All Possible Pseudoknot-Free RNA Secondary Structures. PLoS ONE 7 10.1371/journal.pone.0051204 23300665 PMC3531468

[15] Wuchty S, Fontana W, Hofacker IL, Schuster P (1999) Complete Suboptimal Folding of RNA and the Stability of Secondary Structures. Biopolymers 49: 145–165. 10070264 10.1002/(SICI)1097-0282(199902)49:2<145::AID-BIP4>3.0.CO;2-G

[16] Gruber A, Lorenz R, Bernhart S, Neubock R, Hofacker I (2008) The Vienna RNA Websuite. Nucl Acids Res 36: W70–74. 10.1093/nar/gkn188 18424795 PMC2447809

[17] Zuker M, Sankoff D (1984) RNA secondary structures and their prediction. Bull Math Biol 46: 591–621.

[18] Bompfunewerer AF, Backofen R, Bernhart SH, Hertel J, Hofacker IL, et al (2008) Variations on RNA folding and alignment: lessons from Benasque. J Math Biol 56: 129–144. 17611759 10.1007/s00285-007-0107-5

[19] Proctor JR, Meyer IM (2013) COFOLD: an RNA secondary structure prediction method that takes co-transcriptional folding into account. Nucleic Acids Res 41: e102 10.1093/nar/gkt174 23511969 PMC3643587

[20] Amman F, Bernhart SH, Doose G, Hofacker I, Qin J, et al (2013) The Trouble with Long-Range Base Pairs in RNA Folding. Advances in Bioinformatics and Computational Biology Lecture Notes in Computer Science 8213: 1–11.

[21] Washietl S, Hofacker IL, Stadler PF, Kellis M (2012) RNA folding with soft constraints: reconciliation of probing data and thermodynamic secondary structure prediction. Nucleic Acids Res 40: 4261–4272. 10.1093/nar/gks009 22287623 PMC3378861

[22] Larson NB, Day J, Greenwood A, McPherson A (1998) Refined Structure of Satellite Tobacco Mosaic Virus at 1.8A Resolution. J Mol Biol 277: 37–59. 9514737 10.1006/jmbi.1997.1570

[23] Bleckley S, Schroeder SJ (2012) Incorporating Global Features of RNA Motifs in Predictions for an Ensemble of Secondary Structures for Encapsidated MS2 Bacteriophage RNA. RNA 18: 1309–1318. 10.1261/rna.032326.112 22645379 PMC3383962

[24] Schroeder SJ (2014) Alternative Viewpoints and Alternative Structures for Satellite Tobacco Mosaic Virus RNA. Biochemistry accepted. 10.1021/bi501051k 25320869

[25] Shepherd CM, Borelli IA, Lander G, Natarajan P, Siddavanahalli V, et al (2006) VIPERdb: a Relational Database for Structural Virology. Nucl Acids Res 34: D386–D389. 16381893 10.1093/nar/gkj032 PMC1347395

[26] Rudinger-Thirion J, Lescure A, Paulus C, Frugier M (2011) Misfolded human tRNA isodecoder binds and neutralizes a 3′ UTR-embedded Alu element. Proc Natl Acad Sci U S A 108: E794–802. 10.1073/pnas.1103698108 21896722 PMC3189049

[27] Larson SB, McPherson A (2001) Satellite Tobacco Mosaic Virus RNA: Structure and Implications for Assembly. Curr Opin Struct Biol 11: 59–65. 11179893 10.1016/s0959-440x(00)00166-4

[28] Markham NR, Zuker M (2008) UNAFold: software for nucleic acid folding and hybridization. Methods Mol Biol 453: 3–31. 10.1007/978-1-60327-429-6_1 18712296

[29] Ding Y, Chan CY, Lawrence CE (2006) Clustering of RNA secondary structures with applications to messenger RNAs. J Mol Biol 359: 554–571. 16631786 10.1016/j.jmb.2006.01.056

[30] Ding Y, Chan C, Lawrence C (2004) A statistical sampling algorithm for RNA secondary structure prediction. Nucl Acids Res 31: 7280–7301. 10.1093/nar/gkg938 PMC297010 14654704

[31] Lorenz R, Bernhart SH, Honer Z, Siederdissen C, Tafer H, et al (2011) ViennaRNA Package 2.0. Algorithms Mol Biol 6: 26 10.1186/1748-7188-6-26 22115189 PMC3319429

[32] Forum M (1994) MPI: A message-passing interface standard.

[33] Guo F, Gooding AR, Cech TR (2004) Structure of the Tetrahymena ribozyme: base triple sandwich and metal ion at the active site. Mol Cell 16: 351–362. 15525509 10.1016/j.molcel.2004.10.003

